# Crystal structure of FlgL and its implications for flagellar assembly

**DOI:** 10.1038/s41598-018-32460-9

**Published:** 2018-09-24

**Authors:** Ho Jeong Hong, Tae Hee Kim, Wan Seok Song, Hyun-Jeong Ko, Geun-Shik Lee, Seung Goo Kang, Pyeung-Hyeun Kim, Sung-il Yoon

**Affiliations:** 1 Division of Biomedical Convergence, College of Biomedical Science, Kangwon National University, Chuncheon, 24341 Republic of Korea; 2 Laboratory of Microbiology and Immunology, College of Pharmacy, Kangwon National University, Chuncheon, 24341 Republic of Korea; 3 College of Veterinary Medicine, Kangwon National University, Chuncheon, 24341 Republic of Korea; 4 Department of Molecular Bioscience, School of Biomedical Science, Kangwon National University, Chuncheon, 24341 Republic of Korea; 5 Institute of Bioscience and Biotechnology, Kangwon National University, Chuncheon, 24341 Republic of Korea

## Abstract

Bacteria move toward attractants and away from repellants by rotating their flagellum. The bacterial flagellum assembles through the ordered organization of more than 30 different proteins. Among the diverse flagellar proteins, FlgL forms the junction between the hook and the filament in the flagellum together with FlgK and provides a structural base where flagellin, a filament-forming protein, is inserted for the initiation of filament elongation. However, the functional and structural information available for FlgL is highly limited. To provide structural insights into the cross-linkage between the FlgL junction and the flagellin filament, we determined the crystal structures of FlgL from gram-positive *Bacillus cereus* (bcFlgL) and gram-negative *Xanthomonas campestris* (xcFlgL). bcFlgL contains one domain (D1), whereas xcFlgL adopts a two-domain structure that consists of the D1 and D2 domains. The constant D1 domain of FlgL adopts a rod structure that is generated by four longitudinal segments. This four-segment structure is recapitulated in filament and junction proteins but not in hook and rod proteins, allowing us to propose a junction-filament assembly mechanism based on a quasi-homotypic interaction. The D2 domain of xcFlgL resembles that of another junction protein, FlgK, suggesting the structural and functional relatedness of FlgL and FlgK.

## Introduction

The flagellum enables bacteria to move toward attractants or away from detrimental chemicals and thus plays a key role in the dynamic responses of bacteria to diverse environments^1,2^. Moreover, in the host, the flagellum contributes to the tissue colonization and invasion of pathogenic bacteria and functions as a virulence factor. However, the host senses flagellar proteins as pathogen-associated molecular patterns using innate immune receptors for the activation of the immune response to pathogenic bacteria^2–4^.

**Figure 1:**
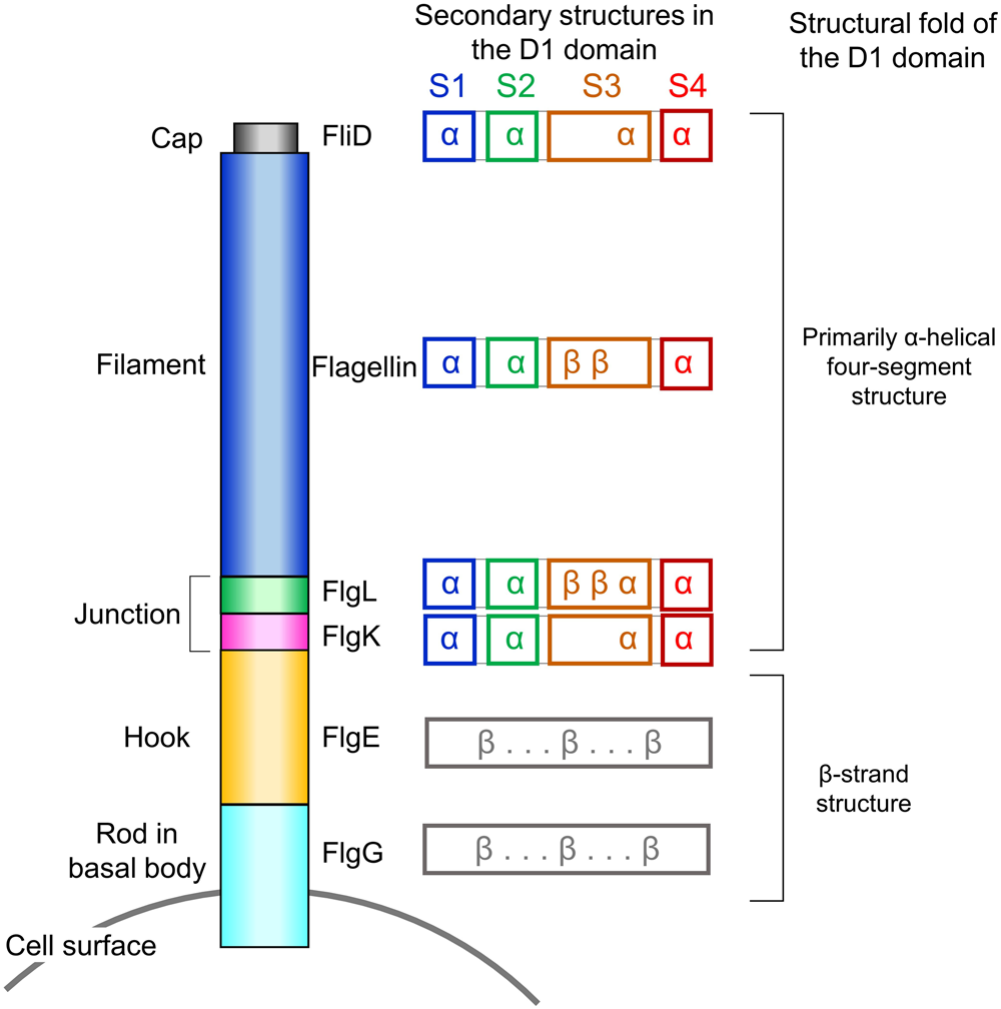
Schematic diagram of the extracellular part of the flagellum. The D1 domains of FlgK, FlgL, flagellin, and FliD commonly adopt primarily α-helical four-segment structures. The secondary structures (α-helix, “α”; β-strand, “β”) that constitute each segment are indicated. In contrast to the D1 domains of FlgK, FlgL, flagellin, and FliD, those of FlgE and FlgG fold into β-strand structures.

The bacterial flagellum is structurally divided into three parts: the basal body, the hook, and the filament^1,5^. The basal body passes through the cell membrane and the cell wall and generates the flagellar rotation force. The basal body is also involved in the export of hook and filament proteins to the extracellular space through the tubular structure of the central rod (Fig. 1). The hook is generated by polymerizing more than 100 copies of the FlgE protein on the distal rod protein FlgG of the basal body and functions as a flexible joint that links the basal body and the filament. The filament is a long extracellular tubule that is elongated through the helical assembly of flagellin. To initiate filament growth, three junction proteins, namely, FlgK (hook-associated protein 1), FlgL (hook-associated protein 3), and the cap protein FliD (hook-associated protein 2), are sequentially placed at the distal end of the hook^6–9^. The flagellin protein is exported from the cytosol through the hollow tubules of the rod and hook and is polymerized to the filament above the FlgL junction layer under the FliD cap^10–12^. Therefore, the extracellular axis of the assembled flagellum forms in the order of FlgG (distal rod protein), FlgE (hook protein), FlgK (junction protein), FlgL (junction protein), flagellin (filament protein), and FliD (cap protein), from the cell surface to the distal end (Fig. 1).

The FlgL and FlgK junction proteins that are located between the FlgE hook and the flagellin filament are indispensable for the formation of the flagellum because both FlgL-deficient bacteria and FlgK-deficient bacteria lack functional flagella and are immotile^13^. In addition to the similar functions and locations of FlgL and FlgK in the flagellum, the *flgL* and *flgK* genes are genetically linked in one operon, and their transcription is simultaneously regulated^6,14^. Moreover, the FlgL and FlgK proteins use the same export chaperone, FlgN, for their delivery to an export gate protein and for the prevention of premature aggregation and proteolysis in the cytosol^15–17^. Recent structural studies of the truncated *Burkholderia pseudomallei* FlgK (bpFlgK) and *Campylobacter jejuni* FlgK proteins revealed that FlgK contains a four-helix bundle structure^18,19^. However, FlgL has not been structurally characterized, and it is thus unknown how FlgL assembles into the upper layer of the junction and links the hook to the filament at the top of FlgK.

In the bacterial flagellum, the hook is directly placed on the rod of the basal body without any junction proteins. However, the hook and the filament are cross-linked through the FlgL and FlgK junction proteins. Based on a structural study of the hook of *C. jejuni*, it was proposed that the junction proteins are required to accommodate the symmetry mismatch between the 11-protofilament organization of the hook and the 7-protofilament organization of the filament^20,21^. However, this symmetry mismatch has been shown to occur only in *C. jejuni* to date. The hooks, rods, and filaments from other species are constituted by the same number (11) of protofilaments, and thus, the biological role of the junction proteins in flagellar assembly remains unclear^12,22,23^.

To investigate the role of FlgL in hook-filament assembly, we have determined the crystal structures of FlgL from gram-positive *Bacillus cereus* (bcFlgL) and gram-negative *Xanthomonas campestris* (xcFlgL). Based on the structural similarity of FlgL to flagellin, we propose that flagellin assembles on top of FlgL in a manner similar to that reported for flagellin polymerization in the flagellar filament.

## Results

### Overall structure of FlgL

**Figure 2:**
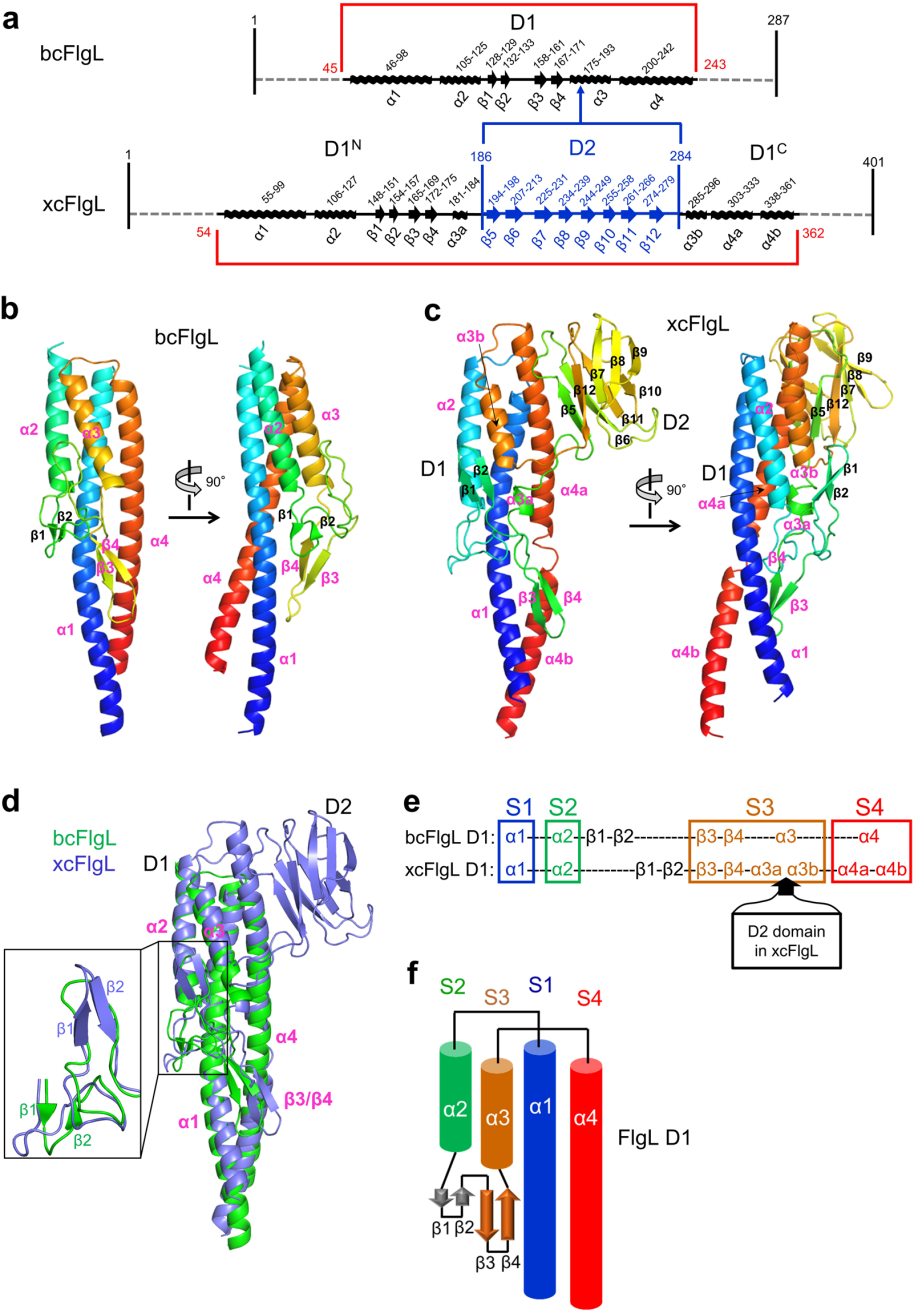
Overall structure of FlgL. (**a**) Schematic diagram that shows the arrangement of domains and secondary structures in bcFlgL and xcFlgL. (**b**,**c**) Structures of bcFlgL (**b**) and xcFlgL^C2^ (**c**). The FlgL structures are shown in rainbow-colored ribbons (N-terminus, blue; C-terminus, red). The secondary structures that bcFlgL and xcFlgL share are highlighted with magenta labels. (**d**) Overlay of the bcFlgL (green ribbons) and xcFlgL (light blue ribbons) structures. The common secondary structures of bcFlgL and xcFlgL are labeled in magenta. The inset shows that the β1 and β2 strands of bcFlgL are positioned differently from those of xcFlgL. (**e**) Conserved four-segment (S1–S4) arrangement in the D1 domains of bcFlgL and xcFlgL. The S1, S2, S3, and S4 segments are represented by the secondary structures of α1, α2, β3/β4/α3, and α4, respectively. (**f**) Topology diagram of the D1 domain in the FlgL structures.

To reveal the FlgL structure, we screened the full-length and truncated proteins of xcFlgL, bcFlgL, *Legionella pneumophila* FlgL (lpFlgL), and *Salmonella enterica* serovar Typhimurium (*S*. Typhimurium) FlgL (stFlgL) for expression, purification, crystallization, and X-ray diffraction. Crystal structures for the truncated bcFlgL and xcFlgL proteins (residues 45–251 and residues 48–364, respectively), which lack ~45 N-terminal residues and ~35 C-terminal residues, were solved by multiwavelength anomalous diffraction (MAD) phasing and single-wavelength anomalous diffraction (SAD) phasing, respectively (Table 1). The bcFlgL structure was refined to 2.2 Å resolution (Fig. 2a,b and Table 2). xcFlgL was crystallized in two different crystal forms, and we obtained two xcFlgL structures (xcFlgL^C2^ structure in space group C2 at 1.9 Å resolution; xcFlgL^H3^ structure in space group H3 at 2.2 Å resolution; xcFlgL structure refers to xcFlgL^C2^ unless specified) (Fig. 2a,c, Supplementary Fig. S1a, and Table 2).

**Table 1:** Data collection and phasing statistics for the crystallographic data obtained from SeMet-FlgL crystals.

	bcFlgL		xcFlgL^H3^
	(Inflection)	(Remote)	(Peak)
**Data collection**			
Space group	C2		H3
Cell parameters			
a (Å)	138.58		178.14
b (Å)	57.89		178.14
c (Å)	67.81		54.85
α (°)	90		90
β (°)	115.03		90
γ (°)	90		120
Wavelength (Å)	0.9795	0.9717	0.9792
Resolution (Å)	30.00-2.00	30.00-2.00	30.00-2.15
Highest resolution (Å)	2.03-2.00	2.03-2.00	2.19-2.15
No. observations	242,149	244,133	376,650
No. unique reflections	33,051	33,306	35,210
R_merge_ (%)^a^	10.5 (60.2)^b^	10.7 (61.6)^b^	11.8 (54.2)^b^
I/sigma(I)	29.7 (3.9)^b^	29.4 (3.8)^b^	42.5 (6.6)^b^
Completeness (%)	100.0 (100.0)^b^	100.0 (100.0)^b^	100.0 (100.0)^b^
Redundancy	7.3 (7.3)^b^	7.3 (7.4)^b^	10.7 (11.4)^b^
**Phasing**			
Resolution (Å)	30.00-2.00		30.00-2.15
No. Se	11		16
Figure of merit			
Prior to density modification	0.33		0.42
After density modification	0.63		0.70

^a^R_merge_ = Σ_hkl_Σ_i_ | I_i_(hkl) − <I(hkl)> |/Σ_hkl_Σ_i_ I_i_(hkl).

^b^Numbers in parentheses were calculated from data for the highest resolution shell.

**Table 2:** Crystallographic statistics of the FlgL structures.

	bcFlgL	xcFlgL^C2^	xcFlgL^H3^
**Data collection**			
Space group	C2	C2	H3
Cell parameters			
a (Å)	138.62	100.18	179.37
b (Å)	57.99	63.70	179.37
c (Å)	67.91	51.28	54.76
α (°)	90	90	90
β (°)	114.80	105.16	90
γ (°)	90	90	120
Wavelength (Å)	1.0000	1.0000	1.0000
Resolution (Å)	30.00-2.20	30.00-1.90	30.00-2.20
Highest resolution (Å)	2.24-2.20	1.93-1.90	2.24-2.20
No. observations	62,981	91,216	114,142
No. unique reflections	23,891	24,596	33,440
R_merge_ (%)^a^	9.6 (43.5)^b^	7.1 (38.6)^b^	8.3 (51.6)^b^
I/sigma(I)	20.2 (4.4)^b^	25.0 (3.3)^b^	21.9 (3.1)^b^
Completeness (%)	95.4 (98.0)^b^	98.7 (97.0)^b^	99.9 (100.0)^b^
Redundancy	2.6 (2.6)^b^	3.7 (3.6)^b^	3.4 (3.4)^b^
**Refinement**			
Resolution (Å)	30.00-2.20	30.00-1.90	30.00-2.20
No. of reflections (work)	22,641	23,167	31,707
No. of reflections (test)	1,214	1,164	1,600
R_work_ (%)^c^	21.2	20.7	20.8
R_free_ (%)^d^	25.0	25.1	25.8
No. atoms			
Protein	2,755	2,256	4,374
Ligands (Zn)	7	—	—
Water	88	149	130
Average B-value (Å^2^)	32.7	31.0	39.3
RMSD bonds (Å)	0.016	0.012	0.012
RMSD angles (°)	1.37	1.21	1.27
Ramachandran^e^ (favored)	99.7%	99.7%	98.7%
(outliers)	0.0%	0.0%	0.0%

^a^R_merge_ = Σ_hkl_Σ_i_ | I_i_(hkl) − <I(hkl)> |/Σ_hkl_Σ_i_ I_i_(hkl).

^b^Numbers in parentheses were calculated from data for the highest resolution shell.

^c^R_work_ = Σ| |F_obs_|−|F_calc_| | / Σ|F_obs_| where F_calc_ and F_obs_ are the calculated and observed structure factor amplitudes, respectively.

^d^R_free_ = as described for R_work_, but for 5% of the total reflections selected at random and omitted from refinement.

^e^Calculated using MolProbity (http://molprobity.biochem.duke.edu).

Although bcFlgL and xcFlgL are orthologous, they structurally differ in the number of domains. The truncated bcFlgL and xcFlgL proteins adopt one-domain (D1) and two-domain (D1-D2) structures, respectively (Fig. 2a–d and Supplementary Fig. S2). The D1 domains of bcFlgL and xcFlgL are superimposable, with the same organization of four α-helices (α1-α4) and one β-hairpin (β3-β4 hairpin), although the β1-β2 hairpin is located in different positions (Fig. 2d–f). The constant D1 domain of FlgL can be defined to consist of four long segments, S1–S4. The S1, S2, and S4 segments are represented by α-helices, namely, the α1, α2, and α4 helices, respectively. The S3 segment comprises the β3-β4 hairpin and the α3 helix. In Dali searches (http://ekhidna2.biocenter.helsinki.fi/dali/), the D1 domains of bcFlgL and xcFlgL were shown to be structurally homologous to those of stFlgL (PDB ID 2D4X, unpublished structure) and lpFlgL (PDB ID 5YTI, unpublished structure), which also exhibit a four-segment structure that contains the α1-α4 helices and the β3-β4 hairpin (Supplementary Fig. S1b,c)^24^.

xcFlgL contains an additional domain, D2, compared to bcFlgL. In the xcFlgL structure, the α3 helix is split into the α3a and α3b helices, between which the D2 domain is inserted, extending from the middle of the S3 segment (Fig. 2c–e). The D2 domain of xcFlgL forms a β-sandwich structure, which consists of a β5-β12-β7-β8-β9 sheet and a β6-β11-β10 sheet (Fig. 2c).

### Structural similarities and differences between the D1 domains of FlgL and other flagellar proteins

**Figure 3:**
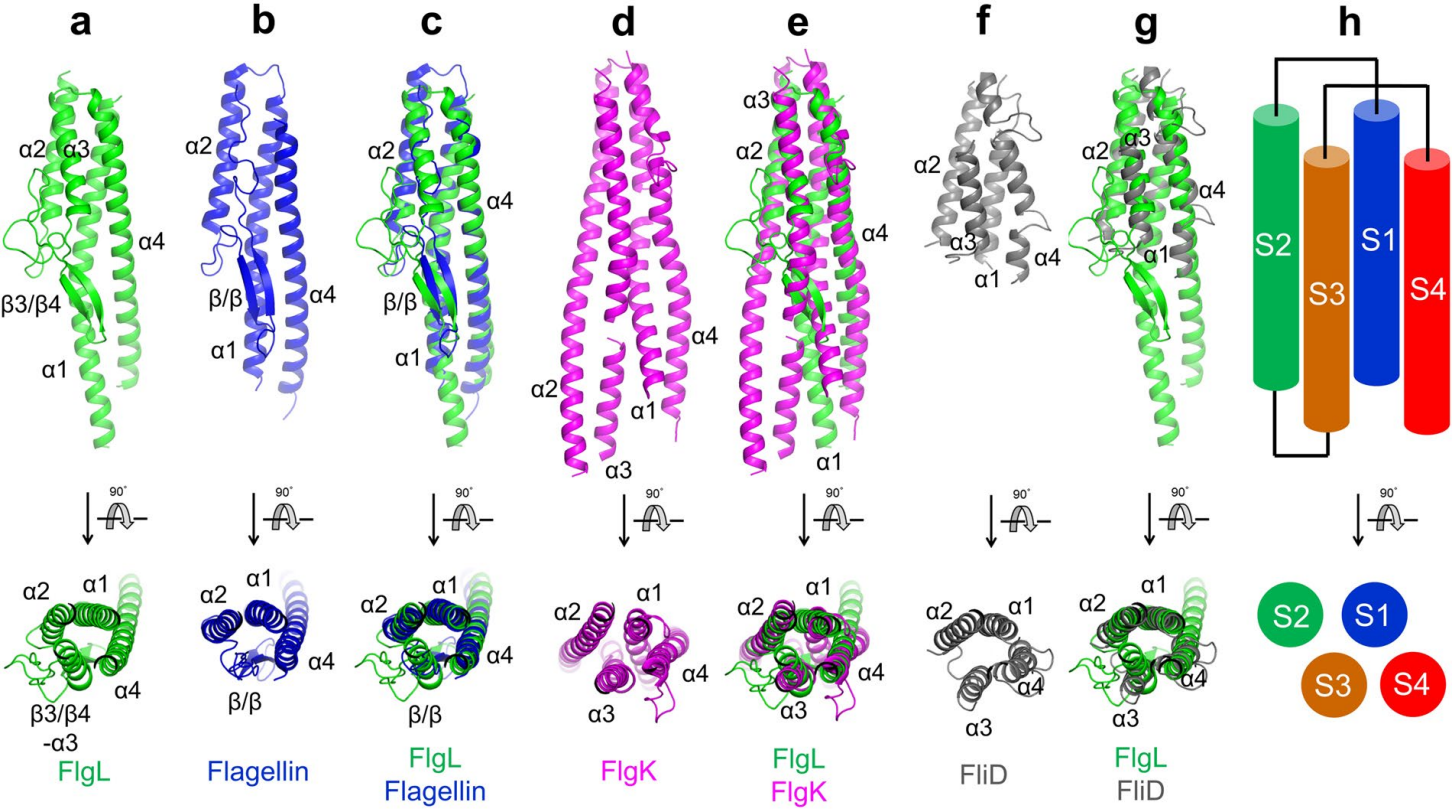
Common four-segment structures found in the D1 domains of FlgL, flagellin, FlgK, and FliD. (**a**) Structure of the D1 domain from bcFlgL (green ribbons). (**b**) Structure of the D1 domain from *S*. Typhimurium flagellin (blue ribbons; PDB ID 1IO1). (**c**) Structural overlay of bcFlgL D1 (green ribbons) and *S*. Typhimurium flagellin D1 (blue ribbons; PDB ID 1IO1). Common secondary structure elements are labeled. (**d**) Structure of the D1 domain from bpFlgK (magenta ribbons; PDB ID, 4UT1). The additional regions of bpFlgK D1 that do not belong to the four-segment structure were removed for clarity. (**e**) Structural overlay of bcFlgL D1 (green ribbons) and bpFlgK D1 (magenta ribbons; PDB ID, 4UT1). Common secondary structure elements are labeled. (**f**) Structure of the D1 domain from *E. coli* FliD (gray ribbons; PDB ID 5H5V). The additional regions of ecFliD D1 that do not belong to the four-segment structure were removed for clarity. (**g**) Structural overlay of bcFlgL D1 (green ribbons) and ecFliD (gray ribbons; PDB ID 5H5V). Common secondary structure elements are labeled. (**h**) Arrangement of the S1–S4 segments shared by the D1 domains of FlgL, flagellin, FlgK, and FliD.

The bcFlgL and xcFlgL structures revealed that the D1 domain of FlgL forms a four-segment structure, irrespective of the bacterial species (Fig. 2e,f). Notably, the four-segment arrangement of FlgL D1 is recapitulated in the D1 domains of other flagellar junction and filament proteins, such as FlgK, flagellin, and FliD, although their sequences and functions are highly diverse (Figs 1 and 3)^10,18,19,25^. In structure overlays, each of the S1, S2, and S4 segments is represented by α-helices that are identical in terms of direction and position. Moreover, the S3 segment is vertically arranged in a similar position although the S3 segment adopts diverse secondary structures (a β-hairpin in flagellin; an α-helix in FliD; a β-hairpin and an α-helix in FlgL; two α-helices in FlgK). Among the flagellar components, the D1 domain of FlgL exhibits the highest structural similarity to that of flagellin, with a root-mean-square deviation (RMSD) value of 1.90 Å between the D1 domains of bcFlgL and *S*. Typhimurium flagellin for 140 Cα atoms (Figs 1 and 3a–c). The D1 domains of FlgL and flagellin both contain a three-α-helix bundle and a β-hairpin, with a similar length for each segment. In addition to flagellin, FlgL D1 structurally resembles FlgK D1 in forming a four-helix bundle although the α2 and α3 helices of FlgL D1 are shorter than those of FlgK (Figs 1 and 3a,d,e). Thus, we conclude that the flagellar junction and filament proteins located after the hook are structurally related to contain the primarily helical four-segment structure in the D1 domain (Figs 1 and 3).

Interestingly, the helical four-segment structure is observed only in the protomers of the flagellar junction and filament but not in other parts below the junction, such as the hook-forming protein (FlgE) and the rod-forming protein (FlgG) (Fig. 1)^20,26^. The D1 domains of FlgG and FlgE share the β-strand structure with a sequence identity of ~40%^20,26^. Therefore, our comparative analysis categorizes the D1 domains of extracellular flagellar building blocks into two structural groups, a β-strand structure in the rod and hook and an α-helical structure in the junction and filament.

The structural similarity between the D1 domains of FlgG and FlgE and between the D1 domains of FlgL and flagellin suggests that FlgE and flagellin assemble on top of FlgG and FlgL, respectively, through quasi-homotypic interactions that mimic the homotypic FlgE-to-FlgE and flagellin-to-flagellin interactions, respectively. The proposed quasi-homotypic interaction provides a clue about the role of FlgK and FlgL in the hook-filament connection. The D1 domain of flagellin adopts a four-segment structure that is completely different from the β-strand structure of the D1 domain of the FlgE hook protein. Therefore, the D1 domain of flagellin would not be able to form a direct quasi-homotypic interaction with the D1 domain of FlgE during flagellar assembly. Instead, flagellin is indirectly cross-linked to FlgE through FlgK and FlgL, which reconcile the structural incompatibility between FlgE and flagellin.

### D2 domain of xcFlgL and its structural similarity to FlgK

**Figure 4:**
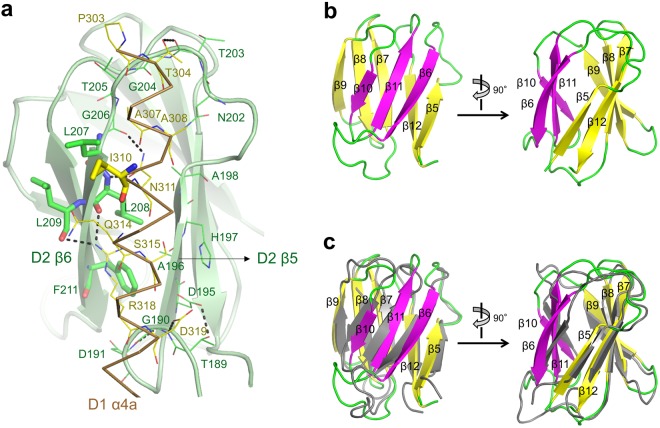
xcFlgL D2 domain and its interaction with the D1 domain. (**a**) D1-D2 interdomain interaction in xcFlgL. The D2 domain is depicted as light green ribbons, and its interdomain interface is represented by green sticks (hydrophobic leucine and phenylalanine residues) and green lines (the remaining residues). The α4a helix of the D1 domain is shown as a brown Cα trace, and its interdomain interface is represented by yellow sticks (hydrophobic isoleucine residue) and yellow lines (the remaining residues). Hydrogen bonds are represented by dotted lines. (**b**) Structure of the xcFlgL D2 domain shown in ribbons. Two β-sheets (β6-β11-β10 and β5-β12-β7-β8-β9) are differently colored (magenta and yellow, respectively). (**c**) Structural similarity between the D2 domains of xcFlgL (yellow, magenta, and green) and bpFlgK (PDB ID 4UT1; gray).

In the xcFlgL structure, the β-sandwich fold of the D2 domain is affixed to the upper part of the helical structure of the D1 domain (Fig. 2c). The β5 and β6 strands of the D2 domain, which constitute one edge of the β-sandwich, interact with the N-terminal region of the α4a helix from the D1 domain (Fig. 4a). Notably, the D1-D2 interdomain orientation and interface are essentially identical in the two crystal structures of xcFlgL (xcFlgL^C2^ and xcFlgL^H3^) despite different crystal contacts, suggesting that the D1-D2 orientation would not change in different environments and would be recapitulated both in solution and in the flagellum. In the xcFlgL structure, the interdomain interface on the D1 domain is mainly polar, and the D1 domain of xcFlgL is therefore highly likely to exist as an independent stable domain without the D2 domain, as observed for bcFlgL. In contrast, because the interdomain interface on the D2 domain of xcFlgL is partially hydrophobic, the structural stability of the D2 domain would be achieved only in the presence of the D1 domain.

The β-sandwich structure of xcFlgL D2, which consists of a three-β-strand sheet and a five-β-strand sheet, is observed in the functionally related FlgK protein (RMSD values of 1.8–1.9 Å with the D2 domain of bpFlgK for ~80 Cα atoms) (Fig. 4b,c)^18,19^. Moreover, the D2 domains of FlgL and FlgK both form accessory structures that are appended to the primarily α-helical D1 domain.

### Common structural features in the terminal regions of FlgL and flagellin

**Figure 5:**
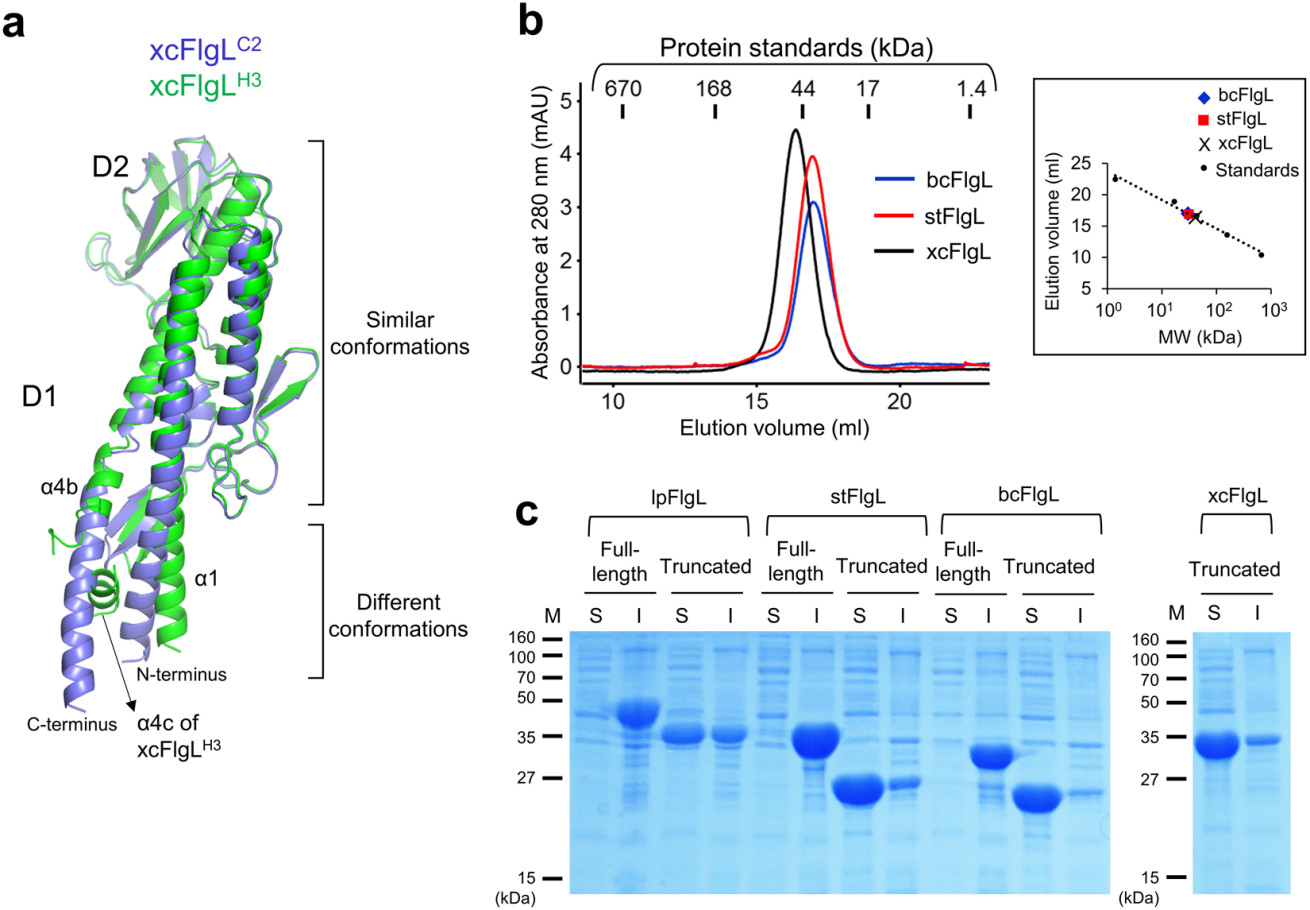
Terminal regions of xcFlgL. (**a**) Different conformations of the N-terminal and C-terminal regions in xcFlgL. The xcFlgL^C2^ (light blue ribbons) and xcFlgL^H3^ (green ribbons) structures exhibit an RMSD value of 0.76 Å for 234 Cα atoms. (**b**) Gel-filtration chromatography profiles for the truncated xcFlgL, stFlgL, and bcFlgL proteins that lack the D0 domain. The truncated bcFlgL, stFlgL, and xcFlgL proteins adopt monomeric forms in solution, with apparent molecular weights of ~30 kDa, ~31 kDa, and ~41 kDa, respectively (theoretical molecular weights, 23.6 kDa, 25.4 kDa, and 33.6 kDa, respectively). The apparent molecular weights of bcFlgL, stFlgL and xcFlgL were estimated using the elution volumes of the gel-filtration standards (vertical lines), as shown in a box. The small differences between the theoretical and apparent molecular weights of the FlgL protein are presumably caused by shape differences between the FlgL protein (elongated shape) and gel filtration standards (globular shape). (**c**) Soluble (“S”) and insoluble (“I”) fractions for the lpFlgL (full-length and truncated), stFlgL (full-length and truncated), bcFlgL (full-length and truncated), and xcFlgL (truncated) proteins that were recombinantly expressed in *E. coli* cells. The expression profile of the full-length xcFlgL protein is not shown because the full-length xcFlgL protein was expressed as an abnormally short fragment. “M” represents protein markers. The full-length gels are shown in Supplementary Fig. S4.

In addition to the common four-segment structure of the D1 domain, FlgL shares the structural features of the terminal regions with flagellin. The N-terminal and C-terminal regions of flagellin were shown to be highly flexible in solution^27^. To be consistent, the terminal regions of the flagellin D1 domain undergo structural rearrangements depending on their environments^12,28^. Our comparative analysis of the two xcFlgL structures (xcFlgL^C2^ and xcFlgL^H3^) indicates that the N-terminal and C-terminal regions of the FlgL D1 domain adopt diverse conformations, as observed for flagellin (Fig. 5a). In the xcFlgL^C2^ structure, the α4b helix runs downwards as one α-helix. However, in the xcFlgL^H3^ structure, the α4b helix is split into two discontinuous α-helices (α4b and α4c) due to a sharp directional change in the helix. Moreover, the N-terminal regions of the α1 helices are tilted in different directions in the two xcFlgL structures. Interestingly, despite the conformational differences, the terminal regions of the FlgL D1 domain have a tendency to form primarily helical structures regardless of crystal contacts.

The N-terminal and C-terminal regions of extracellular flagellar proteins have been structurally defined in FlgG, FlgE, and flagellin and have been shown to form an independent domain, D0^12,20,23^. The D0 domain forms the inner ring of the flagellar tubule and plays a key role in protein polymerization and flagellar assembly. The C-terminal portion of the D0 domain invariably folds into a single α-helix. However, the N-terminal portion of the D0 domain is shaped into two types of structures depending on its length. FlgG and flagellin contain a relatively short N-terminal portion of the D0 domain that adopts a single α-helix. However, the N-terminal portion of the FlgE D0 domain is relatively long and forms a mixed structure of one α-helix and multiple β-strands. The FlgL N-terminal portion that is missing in the bcFlgL structure is relatively short (44 residues), and the terminal regions of FlgL are highly likely to form α-helices based on secondary structure prediction, suggesting that the D0 domain of FlgL adopts a coiled-coil structure that consists of two α-helices, as observed for flagellin. Thus, FlgL structurally mimics flagellin in the D1 domain and presumably in the D0 domain, supporting our proposal that FlgL would recruit flagellin to the junction through quasi-homotypic interactions, as reported for flagellin polymerization in the filament.

Although the D0 domain of FlgL has not been visualized in a structural study, our gel-filtration chromatography and protein expression analyses indicate that the D0 domain modulates the protein solubility of FlgL, as observed for CBLB502, a variant protein of *Salmonella* flagellin, in which the D0 domain was shown to enhance aggregate formation^28^. The gel-filtration chromatography results showed that the truncated bcFlgL, stFlgL and xcFlgL proteins that lack the D0 domain were monomeric in solution (Fig. 5b). Consistently, in the *Escherichia coli* overexpression profile, the truncated proteins of stFlgL and bcFlgL were primarily soluble, and that of lpFlgL was more abundant in the soluble fraction than in the insoluble fraction (Fig. 5c). However, the inclusion of the D0 domain in the recombinant FlgL protein dramatically shifted the protein solubility profile. The full-length proteins of lpFlgL, stFlgL, and bcFlgL were predominantly insoluble, suggesting that the D0 domain of FlgL contributes to aggregation (Fig. 5c). Formation of the unfunctional FlgL aggregate is prevented by the FlgN chaperone, which interacts with the C-terminal region of FlgL in the cytosol^17^. Similarly, the FliS chaperone retains flagellin in a monomeric form by sequestering the C-terminal region of flagellin from aggregation^29^. Taken together, these findings suggest that the terminal regions of FlgL and flagellin exhibit common features in terms of solubility, chaperone binding, and structure.

## Discussion

Our structural analysis demonstrated that bcFlgL and xcFlgL both contain the D1 domain, which forms a primarily helical rod-shaped structure involving four longitudinal segments. Interestingly, the helical four-segment structure of FlgL D1 was also observed in junction and filament proteins but not in other flagellar components located below the junction. The D1 domain of FlgL exhibited the highest structural similarity to the D1 domain of flagellin. Moreover, as observed for flagellin, the terminal regions of FlgL change their structural conformations depending on the environment. Based on the structural resemblance of FlgL to flagellin, we propose that FlgL enables flagellin to assemble on the junction through quasi-homotypic interactions in a mode similar to the homotypic interactions reported for the flagellin-to-flagellin assembly of the filament^12^.

**Figure 6:**
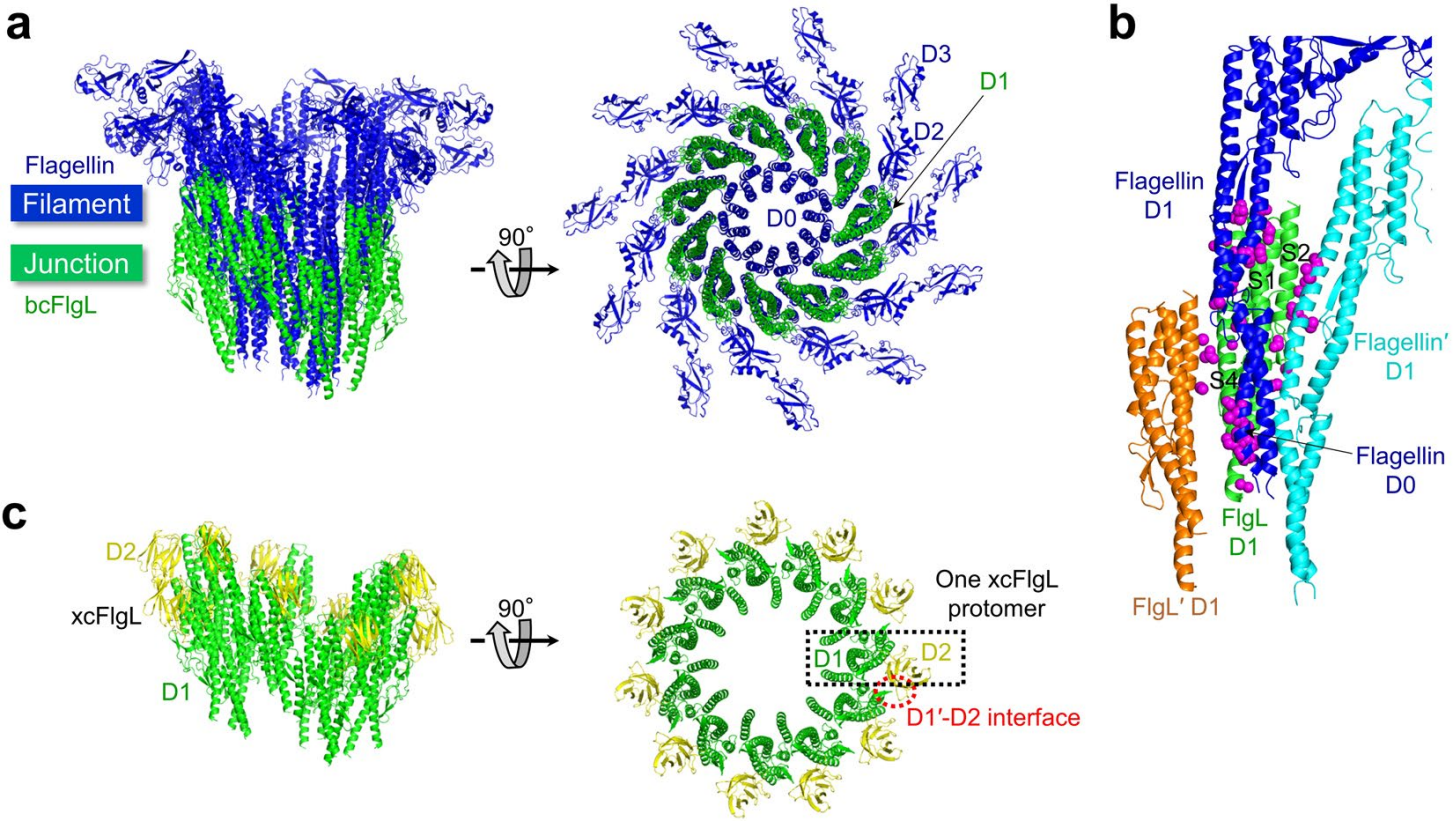
Structural model of FlgL and flagellin assembly in the flagellum. (**a**) FlgL-flagellin model. To build a model that consists of 11 FlgL protomers (green) and 11 flagellin protomers (blue), 22 flagellin protomers that constitute two continuous layers were obtained from the cryo-EM structure of the *S*. Typhimurium filament, and the bottom 11-repeat flagellin layer was overlaid and replaced with 11 bcFlgL D1 domains. *S*. Typhimurium flagellin consists of four domains, D0–D3. The conserved D0 and D1 domains of *S*. Typhimurium flagellin occupy the inner and middle rings of the filament, respectively, and are responsible for inter-flagellin interactions. However, the hypervariable D2 and D3 domains radiate to the outside and do not mediate flagellin polymerization. The structure of the bcFlgL D1 domain is superimposable on that of the flagellin D1 domain and assembles into the middle ring in the flagellum. (**b**) Interactions of FlgL (green ribbons) with its adjacent FlgL molecule, FlgL′ (orange ribbons), and flagellin molecules (blue and cyan ribbons) in the FlgL-flagellin assembly model shown in Fig. 6a. FlgL′, flagellin, and flagellin′ residues that make contact with FlgL are represented by magenta spheres. The S1, S2, and S4 segments of FlgL, which interact with adjacent molecules, are labeled. (**c**) Structural model of the xcFlgL D1 (green) and D2 (yellow) domains in the flagellum. One xcFlgL protomer is boxed in a black dotted rectangle. The binding interface between FlgL D2 and FlgL′ D1 is highlighted with a red dotted circle.

To gain structural insights into the junction-filament assembly of the flagellum, we generated a structural model of the FlgL-flagellin complex in which the FlgL protomers are juxtaposed with the flagellin protomers through quasi-homotypic interactions. The model was built by locating the D1 domains of FlgL in place of the bottom 11-repeat flagellin layer in the flagellar filament of *S*. Typhimurium (Fig. 6a)^12^. The FlgL-flagellin model did not exhibit any major steric clashes between FlgL protomers or between FlgL and flagellin protomers. In the model, FlgL D1 molecules helically assemble into a twisted ring. The D1 domain of FlgL simultaneously makes contact with its adjacent FlgL molecule (FlgL′; the prime indicates the adjacent molecule) and two flagellin molecules (flagellin and flagellin′) via the S1, S2, and S4 segments (Fig. 6b). The lower part of FlgL D1 interacts with the D0 domain of flagellin, and the upper part of FlgL D1 simultaneously binds FlgL′ and the lower parts of the flagellin and flagellin′ D1 domains. The complete fit of the FlgL structure to the flagellin filament suggests that flagellin assembles on FlgL in a mode similar to the homotypic interactions reported for flagellin-to-flagellin polymerization^12^. Thus, we propose that a nascent flagellin molecule would be transported to the top of one FlgL protomer and inserted between two neighboring FlgL protomers under FliD once every two FlgL molecules.

To experimentally support the FlgL-flagellin model, we performed an FlgL-flagellin binding study using full-length *S*. Typhimurium flagellin protein and truncated stFlgL protein lacking the D0 domain. However, no significant interactions were identified by gel-filtration chromatography, native PAGE, and cross-linking (Supplementary Fig. S3). These results suggest that the interaction of the truncated FlgL protein with flagellin is extremely weak and that the FlgL-flagellin interaction might require the D0 domain of FlgL, other flagellar component proteins, or a flagellar framework.

In the FlgL-flagellin assembly model, the D2 domain of FlgL is exposed to the outside and decorates the internal ring composed of the FlgL D1 domains (Fig. 6c). Although the functional role of the xcFlgL D2 domain in the flagellum has not been experimentally determined, the variable D2 domain of FlgL might contribute to the robustness of the junction or the junction-filament assembly. In the FlgL-flagellin assembly model, a small binding interface is observed between FlgL D2 and FlgL′ D1, suggesting that the FlgL D2 domain reinforces the inter-FlgL interactions at the junction (Fig. 6c). This D2-mediated junction reinforcement can be proposed in *B. pseudomallei*. The bpFlgK structure and the *B. pseudomallei* FlgL (bpFlgL) sequence indicate that both bpFlgL and bpFlgK contain a D2 domain that would adopt a similar structure^18^. Thus, bpFlgL is expected to interact with bpFlgK in the flagellum through a quasi-homotypic interaction between the D2 domains of the two proteins. Similarly, the D2 domain of FlgK was proposed to interact with the variable D3 and D4 domains of FlgE^19^. To reveal the functional role of the D1 or D2 domains that we have proposed here, future structural studies of the FlgL-flagellin interaction are required.

## Methods

### Construction of the protein expression vector

The bcFlgL gene (D1 domain; residues 45–251) and the xcFlgL gene (D1 and D2 domains; residues 48–364) were amplified by PCR from the genomic DNAs of *B. cereus* and *X. campestris*, respectively, and cloned into a pET49b expression vector that was modified to express a protein containing an N-terminal His_6_ tag and a thrombin cleavage site^30,31^. The cloned product was transformed into *E. coli* strain DH5α, and the nucleotide sequence of the FlgL expression plasmid was verified by DNA sequencing. For protein expression, the expression plasmid was transformed into *E. coli* strains BL21 (DE3) and B834 (DE3).

### Protein production and purification

The recombinant proteins were overexpressed in *E. coli* BL21 (DE3) cells. The cells were grown in Luria-Bertani (LB) broth at 37 °C to an optical density of 0.6 at 600 nm. Protein overexpression was induced for 18 hours at 18 °C by supplementing the LB medium with 1 mM isopropyl β-D-1-thiogalactopyranoside. Selenomethionine (SeMet)-incorporated bcFlgL and xcFlgL proteins (SeMet-bcFlgL and SeMet-xcFlgL, respectively) were produced in *E. coli* B834 (DE3) cells using nutrient-supplemented M9 minimal medium (Molecular Dimensions) containing 40 μg/ml L-SeMet. The overexpression of SeMet proteins was induced in a manner similar to that used for the native protein.

The cells were pelleted by centrifugation, resuspended in 50 mM Tris, pH 8.0, and 200 mM sodium chloride, and sonicated for cell lysis. The soluble lysate was incubated with Ni-NTA resin (Qiagen) in the presence of 10 mM imidazole at 4 °C for 1 hour for initial purification by Ni-NTA affinity chromatography. The FlgL-bound Ni-NTA resin was packed in an Econo-column (Bio-Rad) and washed with 50 mM Tris, pH 8.0, 200 mM sodium chloride, and 10 mM imidazole. The FlgL protein was eluted from the resin using 250 mM imidazole, 50 mM Tris, pH 8.0, and 200 mM sodium chloride and dialyzed against 20 mM Hepes, pH 7.4. The N-terminal His_6_ affinity tag was removed from FlgL by incubation with thrombin at 18 °C for 3 hours. The resulting FlgL protein was injected into a Mono Q 10/100 column (GE Healthcare) for anion-exchange chromatography and eluted with a linear gradient of sodium chloride (0–500 mM) in 20 mM Hepes, pH 7.4. The oligomeric states of the purified bcFlgL, stFlgL, and xcFlgL proteins were analyzed by gel-filtration chromatography using a Superdex 200 10/300 column (GE Healthcare) in a solution containing 20 mM Hepes, pH 7.4, and 150 mM sodium chloride.

### Crystallization and X-ray diffraction data collection

The crystallization conditions for FlgL were screened by the sitting-drop vapor-diffusion method using the JCSG Core Suites kit (Qiagen). The initial crystallization conditions were optimized to 15% w/v PEG 8000, 0.1 M Hepes, pH 7.4, and 0.2 M zinc acetate for the native bcFlgL protein and to 17% w/v PEG 3350 and 0.3 M magnesium nitrate for the native xcFlgL protein. SeMet-bcFlgL was crystallized in 10% w/v PEG 8000, 0.1 M sodium acetate, pH 5.4, and 0.3 M zinc acetate. SeMet-xcFlgL crystals were obtained in a solution containing 21% w/v PEG 3350 and 0.3 M magnesium nitrate (space group H3) or in a solution containing 19% w/v PEG 6000, 0.1 M Hepes, pH 7.0, and 1.0 M lithium chloride (space group C2). The crystals were transferred into a cryoprotectant solution containing either 25% glycerol or 25% ethylene glycol and then flash-frozen under a cryo-stream at −173 °C. X-ray diffraction was performed at beamlines 5C and 7A of the Pohang Accelerator Laboratory. The X-ray diffraction data were indexed, integrated, merged, and scaled using the HKL2000 program^32^.

### Structure determination and refinement

The initial phases for the SeMet-bcFlgL and SeMet-xcFlgL structures were obtained via MAD phasing and SAD phasing, respectively, using the AutoSol program in the Phenix package^33,34^. The models of SeMet-bcFlgL (space group C2) and SeMet-xcFlgL (space group H3) were built on the MAD and SAD maps, respectively, using the AutoBuild program in the Phenix package and the Coot program^33–35^. The structures of native bcFlgL (space group C2), SeMet-xcFlgL (space group C2; xcFlgL^C2^), and native xcFlgL (space group H3; xcFlgL^H3^) were determined by molecular replacement using a search model of SeMet-bcFlgL (space group C2) or SeMet-xcFlgL (space group H3) using the Phaser program in the CCP4 suite^36^. The final structures of FlgL were obtained through iterative cycles of rebuilding and refinement using the Coot and Refmac5 programs, respectively^35,37^. The structure refinement statistics are shown in Table 2. The bcFlgL structure contains seven Zn^2+^ ions, which are presumably derived from the crystallization solution (Table 2). The Zn^2+^ ions are coordinated by the aspartate, glutamate, or histidine residues of bcFlgL. Some of the Zn^2+^ ions mediate intermolecular interactions in the crystal, suggesting that Zn^2+^ ions stabilize the bcFlgL crystal.

### Structure deposition

The atomic coordinates and the structure factors for FlgL (PDB ID 5ZIY, 5ZJ0, and 5ZIZ) have been deposited in the Protein Data Bank (www.pdb.org).

## Electronic supplementary material

Supplementary Figures 1-4
