# Supplementary material for: Crystal structure of FlgL and its implications for flagellar assembly
Source: Sci Rep. 2018 Sep 24;8:14307. doi: 10.1038/s41598-018-32460-9 (PMC6155364; doi:10.1038/s41598-018-32460-9)
Supplement: Supplementary file 1 — Supplementary Figures 1-4 [file 41598_2018_32460_MOESM1_ESM.pdf]

Supplementary Information

# Crystal structure of FlgL and its implications for flagellar assembly

Ho Jeong Hong[1], Tae Hee Kim[1], Wan Seok Song[1], Hyun-Jeong Ko[2], Geun-Shik Lee[3], Seung Goo Kang[1], Pyeung-Hyeun Kim[4], Sung-il Yoon[1,5] *

[1]Division of Biomedical Convergence, College of Biomedical Science, Kangwon National University, Chuncheon 24341, Republic of Korea

[2]Laboratory of Microbiology and Immunology, College of Pharmacy, Kangwon National University, Chuncheon 24341, Republic of Korea

[3]College of Veterinary Medicine, Kangwon National University, Chuncheon 24341, Republic of Korea

[4]Department of Molecular Bioscience, School of Biomedical Science, Kangwon National University, Chuncheon 24341, Republic of Korea

[5]Institute of Bioscience and Biotechnology, Kangwon National University, Chuncheon 24341, Republic of Korea

* Corresponding author

- Sung-il Yoon (E-mail: sungil@kangwon.ac.kr, Phone: +82-33-250-8385, Fax: +82-33-259-5643, Address: 1 Kangwondaehak-gil, Biomedical Science Building A-204, Chuncheon 24341, Republic of Korea)

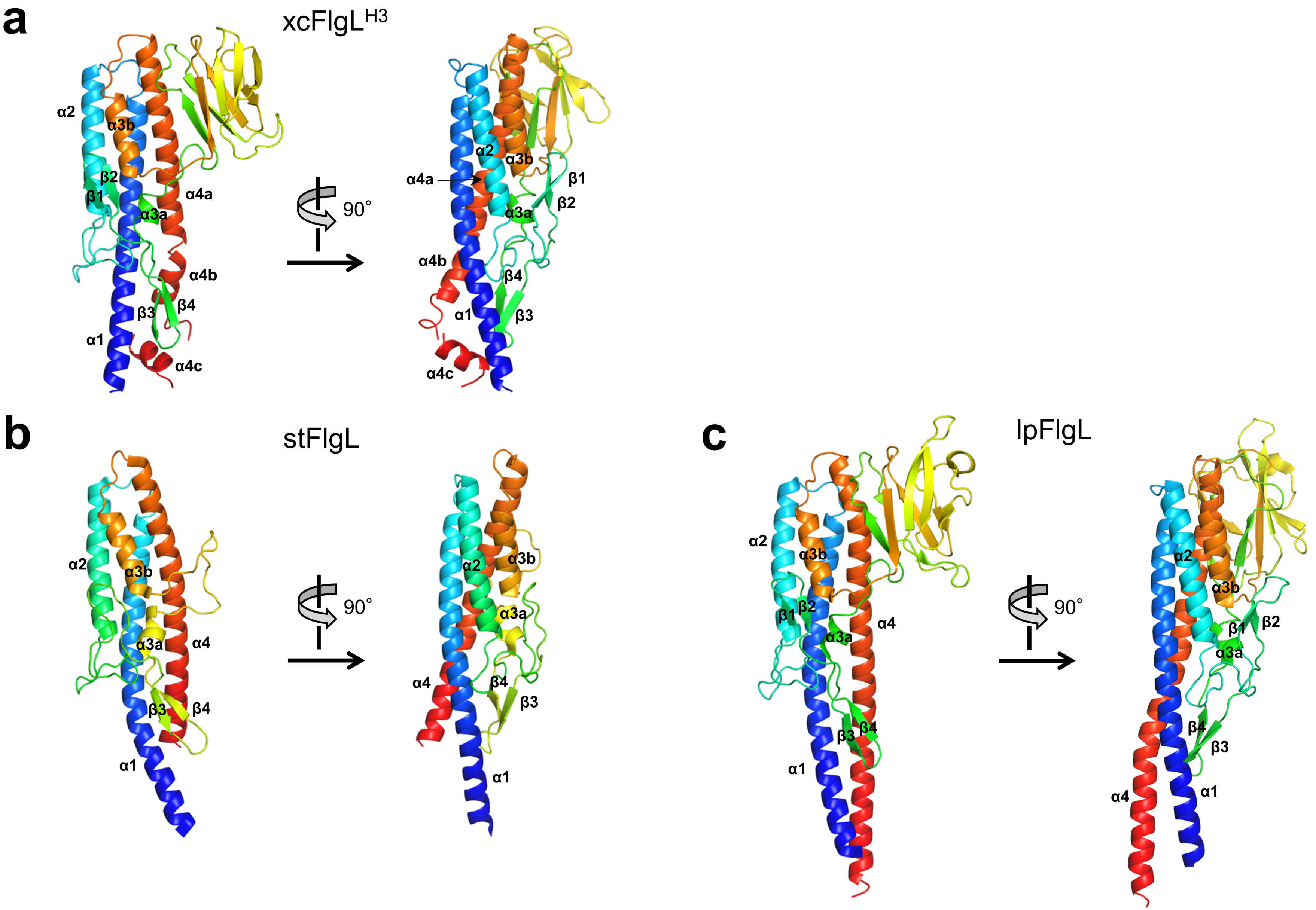


**Supplementary Figure S1. Structures of xcFlgL$^{H3}$ (a), stFlgL (b; PDB ID 2D4X), and lpFlgL (c; PDB ID 5YTI).** The FlgL structures are shown in rainbow-colored ribbons (N-terminus, blue; C-terminus, red). The orientations of the xcFlgL$^{H3}$, stFlgL, and lpFlgL structures are identical to those of the bcFlgL and xcFlgL$^{C2}$ structures in Fig. 2b and Fig. 2c, respectively. stFlgL exhibits an RMSD value of 1.45 Å with bcFlgL for 118 Cα atoms and an RMSD value of 0.90 Å with xcFlgL$^{C2}$ for 130 Cα atoms. lpFlgL shows an RMSD value of 2.87 Å with bcFlgL for 122 Cα atoms and an RMSD value of 1.37 Å with xcFlgL$^{C2}$ for 211 Cα atoms.

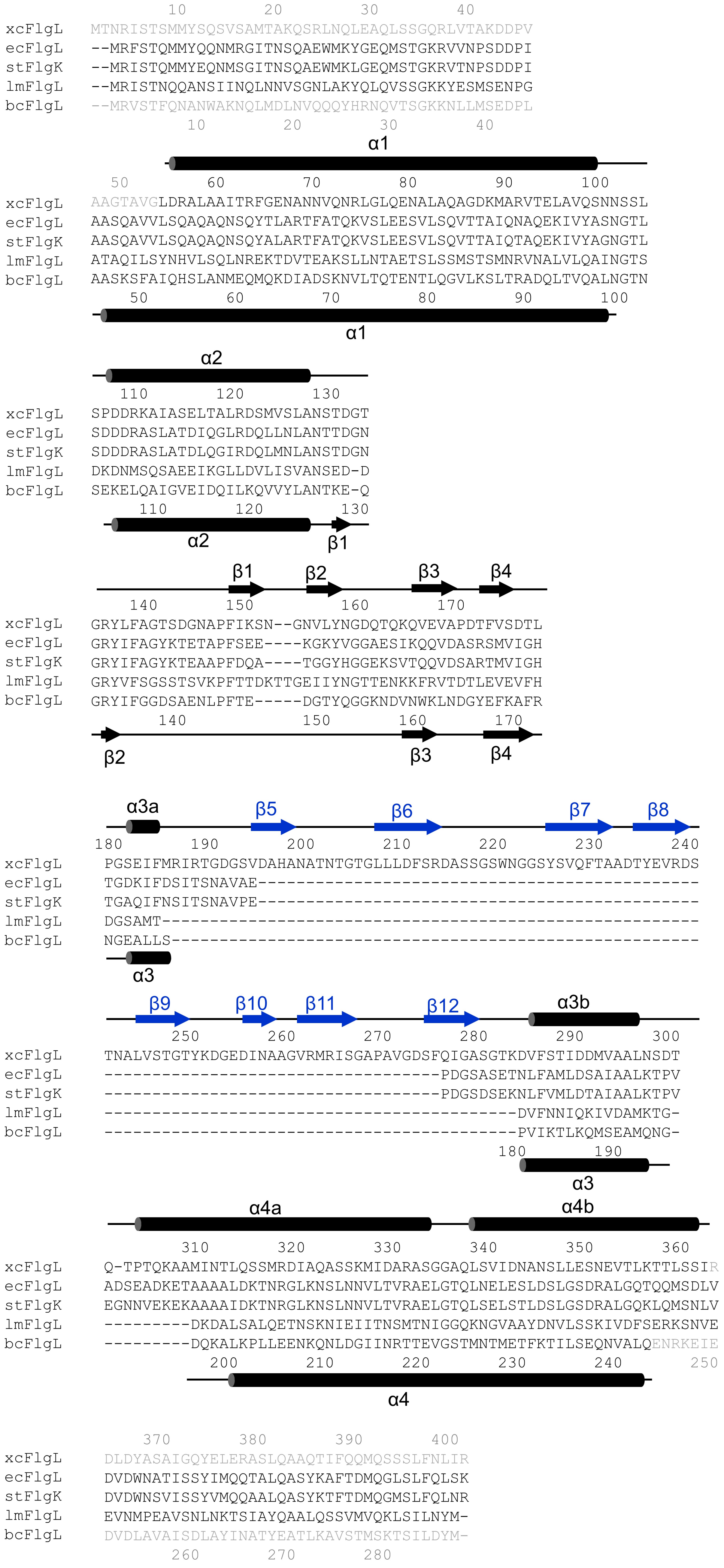


**Supplementary Figure S2. Sequence alignment of xcFlgL, *E. coli* FlgL (ecFlgL), stFlgL, *Listeria monocytogenes* FlgL (lmFlgL), and bcFlgL.** The secondary structures (D1 domain, black; D2 domain, blue) of xcFlgL and bcFlgL are shown above the xcFlgL sequence and below the bcFlgL sequence, respectively, as rods (α-helices) and arrows (β-strands). The N-terminal and C-terminal sequences of xcFlgL and bcFlgL that were not structurally defined are shown in gray.

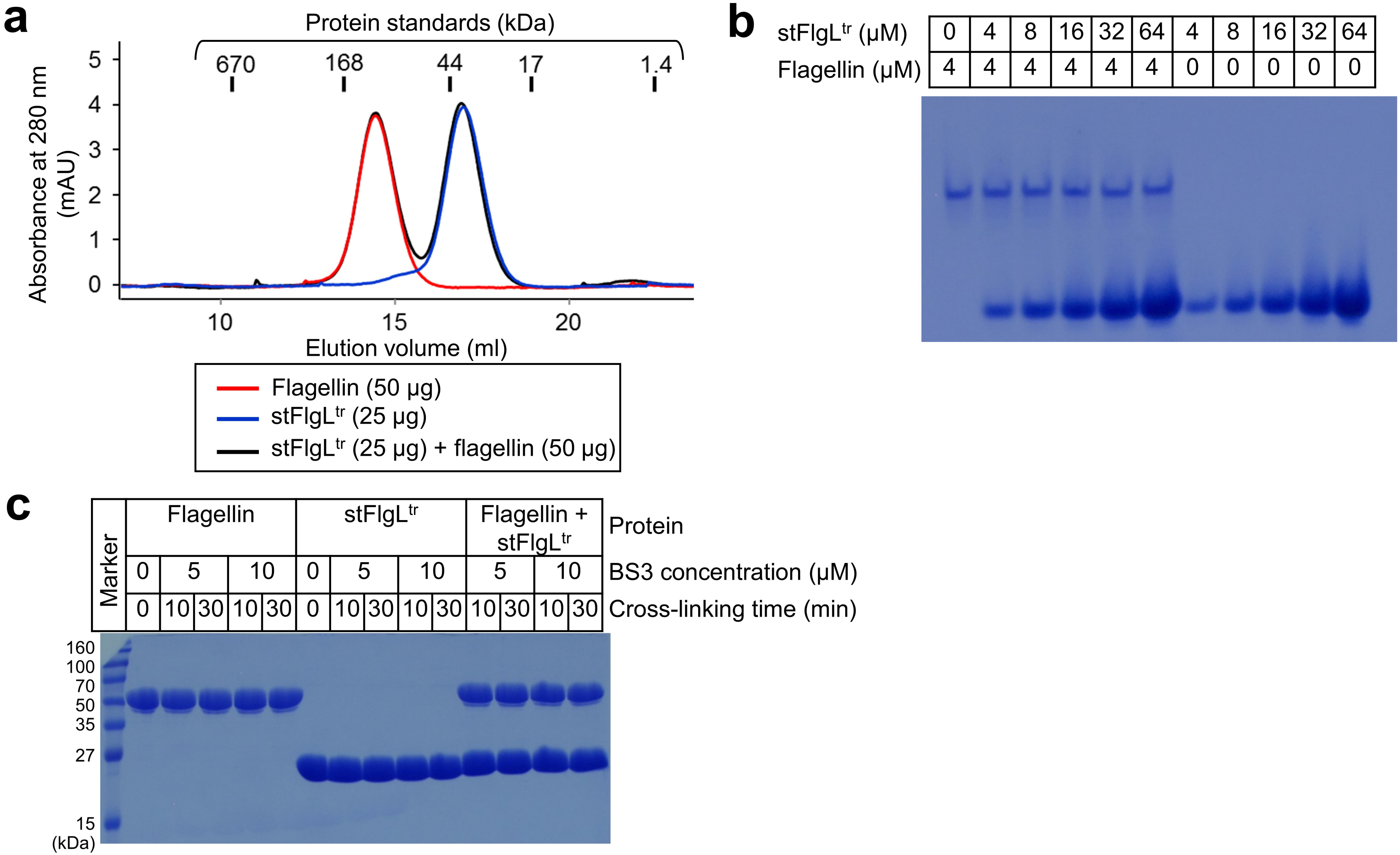


**Supplementary Figure S3. Binding analyses of full-length *S.* Typhimurium flagellin protein and truncated stFlgL protein (stFlgL$^{tr}$) lacking the D0 domain.** (**a**) Gel-filtration chromatography. Protein samples were prepared by incubating proteins at 18°C for 1 hour in 20 mM Hepes, pH 7.4, and 150 mM NaCl, and gel-filtration chromatography was then performed using a Superdex 200 10/300 column with 20 mM Hepes, pH 7.4, and 150 mM NaCl. (**b**) Native PAGE. Protein samples were prepared by incubating proteins at 18°C for 1 hour in 20 mM Hepes, pH 7.4, and 150 mM NaCl, and native PAGE was then performed using a 6% polyacrylamide gel for 90 minutes at 100 V. (**c**) Cross-linking. Proteins were cross-linked using bis(sulfosuccinimidyl)suberate (BS3) at 18°C in 20 mM Hepes, pH 7.4, and 150 mM NaCl and then analyzed by SDS-PAGE.

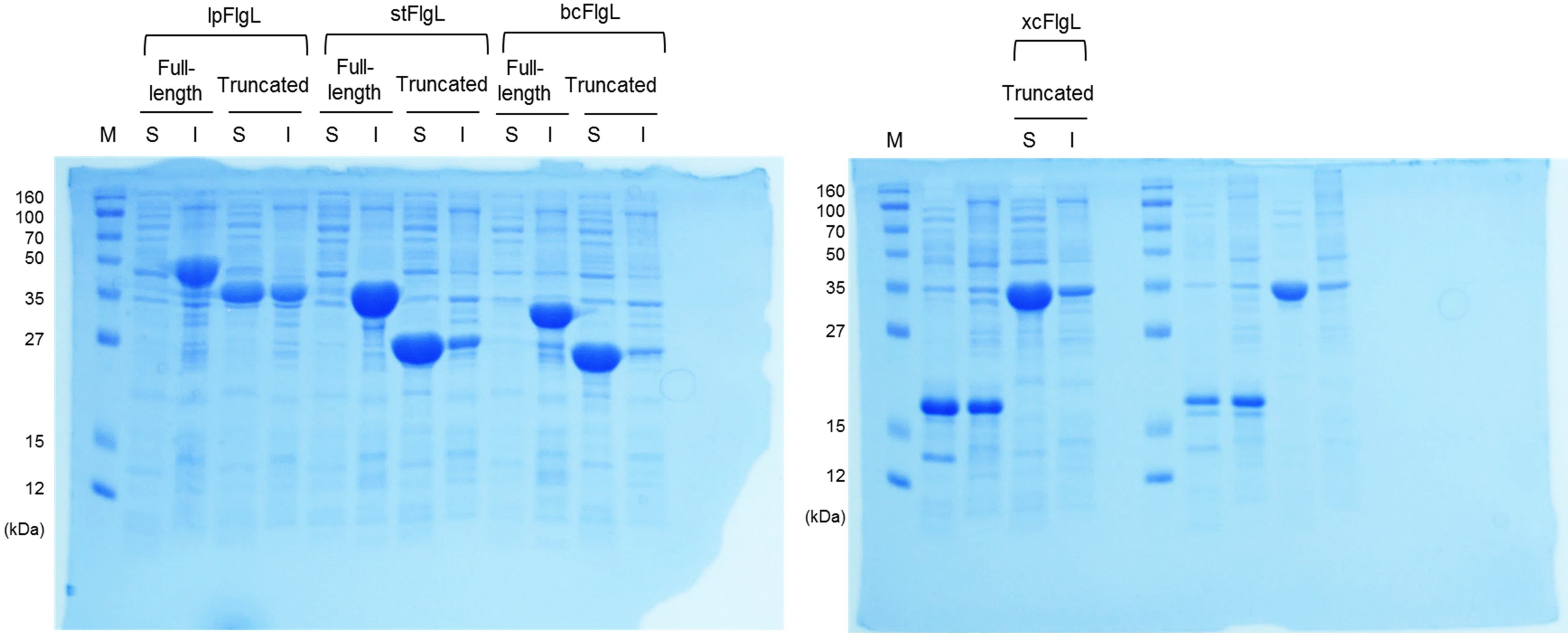


**Supplementary Figure S4. Full-length gels of Fig. 5c.**
